# Pullulan-Based Nanoparticle-HSA Complex Formation and Drug Release Influenced by Surface Charge

**DOI:** 10.1186/s11671-018-2729-5

**Published:** 2018-10-10

**Authors:** Liming Yuan, Yiting Cao, Qian Luo, Wenyu Yang, Xiaofeng Wu, Xiaoping Yang, Di Wu, Siyuan Tan, Ge Qin, Jia Zhou, Yue Zeng, Xinghua Chen, Xiaojun Tao, Qiufang Zhang

**Affiliations:** 1 Department of Pharmacology, Hubei University of Medicine, Shiyan, 442000 Hubei China; 2 Key Laboratory of Study and Discovery of Small Targeted Molecules of Hunan Province, School of Medicine, Hunan Normal University, Changsha, 410013 China

**Keywords:** Nanoparticles, Human serum albumin, Drug release, α-Helix, Binding constant

## Abstract

The nanomaterial composition of nanoparticles and their protein adsorption in the blood is of great significance in the design of drug-loaded nanoparticles. To explore the interaction between the different surface components of nanoparticles (NPs) and protein, we synthesized three kinds of pullulan NP polymers: cholesteric hydrophobically (CH) modified pullulan (CHP), CH-modified animated pullulan (CHAP), and CH-modified carboxylated pullulan (CHSP). Pullulan NPs were prepared by the dialysis method. Dynamic light scattering was used to determine the charge and size of the three NPs. The size of NPs was altered by the number of charge groups when polymers contain the same degree of cholesterol substitution. The zeta potentials were + 12.9, − 15.4, and − 0.698 mV for CHAP, CHSP, and CHP, respectively, and the dimensions were 116.9, 156.9, and 73.1 nm, respectively. Isothermal titration calorimetry was used to determine the thermodynamic changes of NPs with different surface charge, and the effect of human serum albumin (HSA) on the titration was investigated. The changes of enthalpy and entropy demonstrated an interaction between NPs and HSA; the binding constant (*K*_b_) for CHSP, CHP, and CHAP was 1.41, 27.7, and 412 × 10^4^ M^−1^, respectively, with the positive charge for CHAP–HSA, uncharged for CHP–HSA, and negative charge for CHSP–HSA complex. Fluorescence and circular dichroism spectroscopy were used to determine the protein structure change after the complexation between NPs and HSA. The NP and HSA complexation is a complicated process composed of protein α-helical content reduction and the peptide chain extension; CHP NPs had the largest reduction in HSA α-helical content. The drug release rates of all compounds of NP and HSA were significantly lower than those of free drug and drug-loaded NPs after 48 h. The highest and lowest rates were observed in CHSP–HSA and CHP–HSA, respectively. The drug release was significantly influenced by the adsorption of HSA on NPs, and the size and surface charge of NPs played an important role in this process.

## Background

Nano-drug delivery system, such as nanoparticles (NPs) loaded with small molecular antitumor drugs, has sustained and controlled drug release properties as well as targeting effect. Targeted therapy with NPs has become a focus in treating tumors, because they can significantly reduce the side effects of drugs and improve drug efficacy [1–3].

Drug-loaded NPs need to pass through three paths to reach the target site, i.e., blood circulation, tissue to cell pathway, and intracellular movement [4–6]. They also need to overcome the vascular barrier to reach the targeted tissue, then the cell membrane barrier to reach targeted cell [7, 8]. Protein adsorption and exchange are involved in all NPs’ pathways. Finally, protein-adsorbed NPs reach the target cells and release the drugs [1, 9].

The high-abundance proteins such as human serum albumin (HSA), lipoproteins, and globulin are usually adsorbed on the surface of drug-loaded NPs, thus changing the in vivo release behavior and targeting sites of NPs [10]. The number and type of adsorbed proteins are closely related to the concentration of proteins in plasma and the affinity of NPs [11]. The greater the plasma protein concentration, the greater the surface adsorption of the NP [12]. A protein with high affinity can replace the one with weak affinity [13]. Therefore, the surface of the NP is occupied by the protein with high concentration and strong affinity, forming a nano-protein crown [14]. Nano-protein crowns are indispensable for the in vivo function of NPs [15]. For example, if the surface is modified with polysorbate, NPs can deliver the drug through the blood–brain barrier to brain tissue [16]; hydrophobic-modified polysaccharide nanomaterials can interact with HSA in the body, thereby enhancing the control of drug release [17].

The uptake of NPs by cells is affected by a variety of factors such as the physical and chemical properties of NPs, nano-drug concentration, protein adsorption, and cell adhesion [18]. The types and quantities of protein adsorption affect the function of NPs, including drug release control and targeting. As well, the physical and chemical properties of NPs, such as particle size, charge, and surface hydrophobicity, affect the adsorption of protein [19]. The nature of the NP decides its fate during the in vivo process [20]. Amphiphilic macromolecular materials such as hydrophobically modified polysaccharide polymers can be self-assembled into nano-sized particles. The size of the NP plays an important role in its functions of targeting and drug release control [21]. The hydrophobic group in the polymer is a driving force for the formation of the nuclear structure of the NP. The greater the degree of substitution of the hydrophobic group, the smaller the NP [22]. Polymer materials with carboxyl groups, amino groups, and their derivatives are involved in NP self-assembly, so they affect the size of NPs and provide surface charge for easily attaching to the protein with opposite charge [23]. NPs with different surface charges have different ability for protein adsorption and different biological functions [24]. Therefore, we need to explore the interaction between the different surface components of NPs and protein.

HSA is the most abundant protein in the blood. It is essential for the transport, distribution, and metabolism of foreign and endogenous substances. Many small-molecule drugs enter the body and form HSA–drug adsorbents in blood transport, which changes the pharmacological effects of drugs [25]. Drug-loaded NPs are combined with HSA after entering the body; because of the complex structure of NPs, the characteristics of adsorption differ from small-molecule HSA combinations [26]. For instance, adsorption of small-molecule drugs to HSA molecules is rapid; however, HSA adsorption to NPs is slow and complex [27].

CHP nanoparticles as a drug carrier had been studied for a long time, which had showed excellent nanomaterials for drug delivery [28, 29]. In a previous experiment, we studied the interaction between HSA and pullulan NPs with different degrees of cholesterol substitution, cholesteric hydrophobically (CH) modified pullulan (CHP), and found mainly two processes: HSA rapidly attached to the NP surface and then slowly inserted into the hydrophobic core of NPs [30]. Hydrophobic interactions played a major role in the formation of CHP–HSA complexes [31]. The hydrophobicity and shell–core structure of the particles were mainly responsible for the changes in albumin conformation during the NP and HSA interaction [30].

In this study, we manufactured three NPs, CHP, CH-modified animated pullulan (CHAP), and CH-modified carboxylated pullulan (CHSP). Their structure and properties were characterized with Fourier-transform infrared (FTIR) and NMR, and their sizes and potentials were determined by dynamic light scattering (DLS). Isothermal titration calorimetry (ITC) and fluorescence spectroscopy were used to investigate interaction characteristics of the NP–HSA complexes and the effects of the three kinds of NPs on HSA structure. We reveal the effects on drug release with the properties of the NP–HSA complexes, which is vital for the future application of drug delivery system.

## Methods

### Materials

HSA was purchased from Sigma Aldrich (St. Louis, MO, USA). *N*,*N*-Imidazole was from Shanghai stock solution biotechnology (Shanghai). Ethylenediamine, succinic anhydride was provided by Tianjin Star Chemical Reagent (Tianjin). All other chemical reagents were of analytical grade and were from Changsha Huicheng Co. (Changsha, China).

### Synthesis of CHP, CHSP, and CHAP

#### Synthesis of CHP

**Fig. 1:**
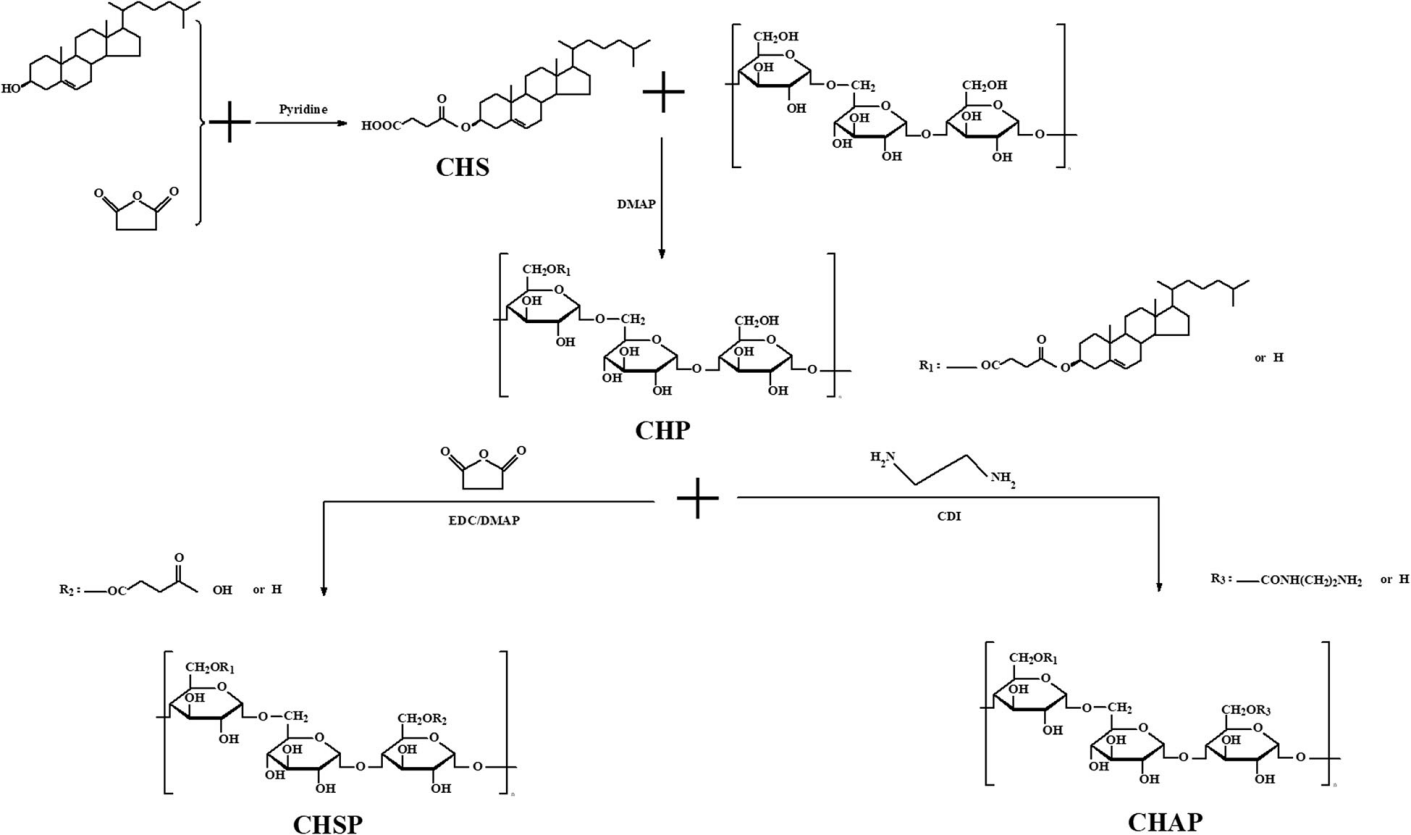
Synthesis of CHP, CHSP, and CHAP polymers

Cholesterol succinate (CHS) was synthesized as previously described [32]. An amount of 2 g pullulan polysaccharide was dissolved in 10 mL dehydrated dimethyl sulfoxide (DMSO) solution. Then, 1.06 g CHS, 0.505 g EDC•HCl, and 0.268 g DMAP were dissolved in an appropriate amount of DMSO solution. The above two groups of reagents were mixed and activated at room temperature for 1 h then incubated in a heated oil bath at 50 °C for 48 h. After the reaction was stopped and cooled to room temperature, an appropriate amount of anhydrous ethanol was added and the white solid was precipitated by stirring and obtained by repeated suction filtrations. The products were washed with an appropriate amount of anhydrous ethanol, ethyl ether, and tetrahydrofuran then dried in a blast dryer at 50 °C to become a white solid (Fig. 1).

#### Synthesis of CHAP

An amount of 1.80 g CHP and 1.00 g *N*,*N*-diimidazole were dissolved in 100 mL DMSO. After heating and stirring in a 50 °C oil bath for 4 h, 3.60 g ethylenediamine was added followed by further heating and stirring for 24 h. When the reaction liquid cooled to room temperature, it was dialyzed in a 4000-interception dialysis bag with double distilled water for 1 day then lyophilized to obtain a light-yellow solid that was the product of hydrophobically modified animated pullulan.

#### Synthesis of CHSP

An amount of 1.80 g CHP was dissolved in 100 mL dehydrated DMSO, then 0.5 g succinic anhydride and 0.05 g 4-dimethylaminopyridine (DMAP) were dissolved in 10 mL DMSO activated for 1 h; after heating and stirring in a 50 °C oil bath for 20 h, the reaction was stopped. When the reaction liquid cooled to room temperature, it was placed in an appropriate amount of anhydrous ethanol and stirred to precipitate a white solid. The white solid was washed several times with an appropriate amount of anhydrous ethanol, diethyl ether, and tetrahydrofuran and dried in a blast dryer at 50 °C. The obtained product was hydrophobically modified carboxylated pullulan polysaccharide.

### FTIR and NMR Spectroscopy

The FTIR spectra for CHP, CHSP, and CHAP were obtained as KBr pellets for FTIR spectroscopy (Nicolet NEXUS 470-ESP, Thermo Fisher Scientific, Waltham, MA, USA). The chemical structures of CHP, CHSP, and CHAP were confirmed by 500 MHz ^1^H-NMR, with DMSO-d6 as the solvent. The degree of substitution of cholesterol in CHP polymers was determined by the alpha-1,4 and alpha-1,6 glycosidic bond and methylene peak area.

### Preparation and Characterization of NPs

CHP, CHSP, and CHAP NPs were prepared by the dialysis method [33]. Briefly, CHP, CHSP, and CHAP were dissolved in 10 mL DMSO. To form NPs, the mixture solution was injected in a dialysis bag for 24 h to eliminate the DMSO. The solution of CHP, CHSP, and CHAP NPs was screened with a membrane filter (pore size 0.45 m, Millipore, Boston, MA, USA) to remove the larger aggregated CHP, CHSP, and CHAP NPs. The size distribution and zeta potential of the obtained particles were determined by DLS (Zetasizer 3000 HS, Malvern Instruments, Malvern, UK) at 11.4 V/cm, 13.0 mA.

### ITC

A certain concentration of HSA solution was dripped onto the CHP, CHSP, and CHAP NP solutions, and the change in heat was measured by ITC (VP-ITC, Microcal, Northampton, MA, USA). An amount of 0.9 mM HSA was injected into 0.01 mM CHP, CHSP, and CHAP NP titration cells, for titrating 20 times. The first drop was 2 μL, and the reaction time was 180 s; the remaining drops were 10 μL per drop and the reaction time was 210 s, and the temperature was set at 25 °C. The thermodynamic parameters and connection curves were obtained with 28 times of titration.

### Fluorescence Spectroscopy

HSA and CHP NPs were mixed at a molecule ratio of HSA to CHP of 3.6:1 to prepare CHP–HSA, CHSP–HSA, and CHAP–HSA mixtures. The obtained mixtures were placed in 2-mL EP tubes and shaken at 20 rpm at 25 °C for 24 h. Fluorescence spectra and fluorescence intensity (FI) of free HSA and the NP-bound HSA were recorded by fluorescence spectrophotometry (Shimadzu RF-4500, Japan). The tryptophan chromophore in the HSA molecule was excited at 280 nm, and emission spectra were recorded at 290 to 450 nm. Excitation and emission slit widths were 5 and 12 nm.

Seven NP solutions at different concentrations were mixed with HSA solution. The mixed solutions were transferred to 2-mL EP tubes for a 9-h reaction. The obtained samples were collected to measure fluorescence spectra at wavelength 290–450 nm. The fluorescence spectra of pure HSA solution were used as a reference to determine binding constants according to Stern–Volmer analysis. Fluorescence quenching data were analyzed by using the improved Stern–Volmer equation [34]:

$$ {F}_0/\left({F}_0-F\right)=1/{f}_{\mathrm{a}}+1/\left({f}_{\mathrm{a}}{K}_{\mathrm{q}}\left[Q\right]\right) $$

where *K*_q_ is the quenching constant of Stern–Volmer, *F*_0_ and *F* are fluorescence intensities at 342 nm in absence and presence of quencher, and [*Q*] is the concentration of quencher.

### Circular Dichroism Analysis

CHP–HSA complexes were prepared through two different ways. The first one (complex I) was prepared by simply mixing HSA and CHP solutions. The second one (complex II) was kept in 2-mL EP tubes, which were set in shaking table with 20 rpm for 12 h at 25 °C. Circular dichroism (CD) spectra for free HSA and NPs added to protein were recorded at wavelength 200–250 nm by using a CD spectrometer (JASCO J-810, Japan) at 37 °C with a 0.1-cm cuvette cell. The concentration of HSA was 1.0 mg/mL in all samples. The relative α-helix content in HSA was calculated as follows [35]:

$$ \left[{\theta}_{208}\right]=\frac{\theta M}{10 CL{N}_{\mathrm{r}}} $$

where *θ*_208_ is the mean residue ellipticity (deg cm^−2^ dmol^−1^) at 208 nm, *θ* is the ellipticity, *M* is molecular weight of HSA, *C* is the concentration of HSA (mg/mL), *L* is length of the cuvette cell (cm), and *N*_r_ is the number of amino acids in the HSA molecule.

### Drug Release In Vitro

Mitoxantrone (MTO)-loaded NPs were prepared by a dialysis method [36]. The standard curve for mitoxantrone was acquired by UV spectrophotometry. Drug loading and encapsulation efficiency were calculated as described [33]. MTO release was studied in vitro by dialysis in phosphate buffered saline. Briefly, the solution of MTO-loaded NPs (2 mg/mL) was placed into visking dialysis tubing and dialyzed against the release media at 37 °C in an air-bath shaker at 50 rpm. At predefined times, the release media was collected and the fresh release media was added. The released amount of MTO was determined by UV spectrophotometry (UV-384 plus, Molecular Devices, USA) at 608 nm. The accumulated release percentage (*Q*%) was calculated as described previously [37]. A certain amount of HSA solution (0.1 mg/mL) was added to the dialysis tube to determine the drug release of three types of NPs.

## Results

### Characterization of CHP, CHSP, and CHAP Polymers

#### FTIR Spectra

**Fig. 2:**
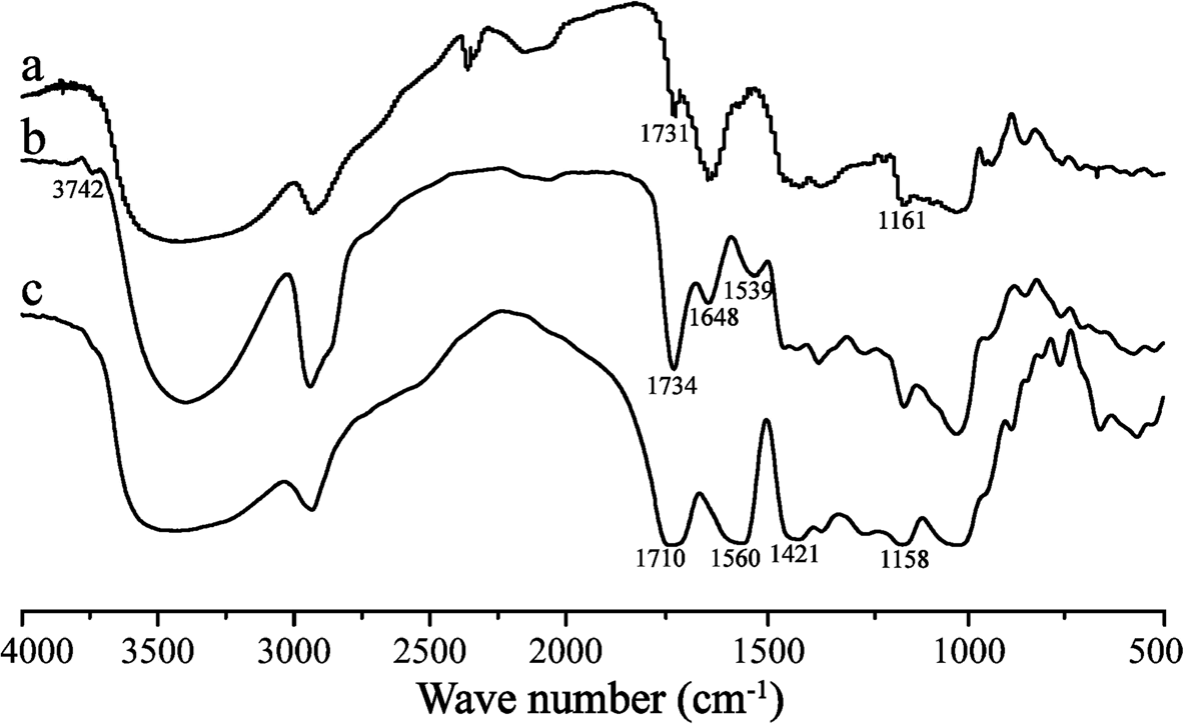
FTIR spectra of CHP (a), CHAP (b), and CHSP (c)

Figure 2 shows the FTIR spectra for CHP, CHSP, and CHAP. The data for CHP spectra were 1731 cm^−1^ (–C=O stretching vibration peak) and 1161 cm^−1^ (–C=O stretching vibration peak). This result demonstrates the formation of ester bonds on pullulan, indicating that the CHP had been successfully synthesized.

As compared with CHP spectra, data for CHAP spectra were 1648 cm^−1^ (–C=O vibration absorption peak), 1734 cm^−1^ (–C=O vibration absorption peak), 1539 cm^−1^ (–N–H bending vibration peak), and 3742 cm^−1^ (–NH_2_ stretching vibration peak). According to these characteristic peaks, there were amide bonds on CHP, and CHAP was successfully synthesized by the esterification reaction.

As compared with CHP spectra, data for CHSP spectra were 1710 cm^−1^ (–C=O stretching vibration peak), 1158 cm^−1^ (–C=O stretching vibration peak), 1560 cm^−1^(double –C=O coupling vibration peak), and 1421 cm^−1^ (–O–H bending vibration peak). This shows that there were carboxyl groups on CHP and part of them became salts.

#### ^1^H NMR

**Fig. 3:**
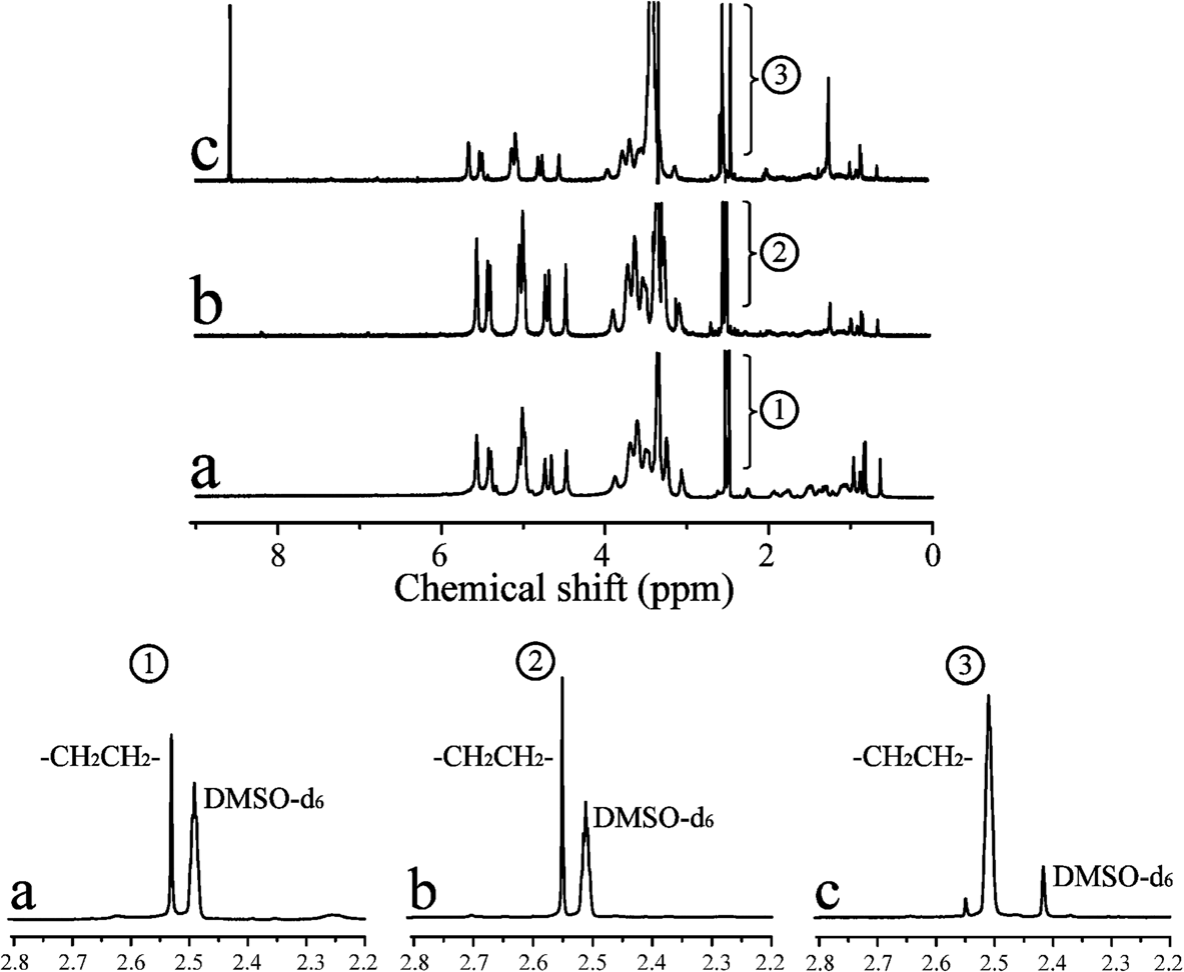
^1^HNMR spectra for CHP (a), CHSP (b), and CHAP (c) NPs

Figure 3 shows the ^1^H NMR spectra for CHP, CHSP, and CHAP. A total of 0 to 2.40 ppm belonged to the hydrogen signal of cholesterol, which demonstrated the successful synthesis of CHP. The characteristic peaks of DMSO-d*6* and methylene (–CH_2_CH_2_–) showed signals at 2.49 and 2.53 ppm, respectively. As compared with CHP, CHAP showed signals at 8–9 ppm, which belonged to the amino group and proved that ethylenediamine was grafted onto CHP. The degree of substitution of cholesterol per 100 glucose units in CHP could be calculated by the ratio of methylene protons to sugar proton with the following equation [38]:

$$ \mathrm{DS}=\frac{A_{\partial 2.53}}{4\left({A}_{\partial 4.74}+{A}_{\partial 5.01}\right)} $$

where *A*_δ2.53_ is the spectrum area under the characteristic absorption peak of methylene (hydrogen), and *A*_δ4.74_ and *A*_δ5.01_ are the spectrum area under the characteristic absorption peaks for alpha-1,6 and alpha-1,4 glycosidic bonds, respectively.

From the ^1^H NMR spectra in CHP, the degree of substitution of cholesterol succinate (CHS) was 4.50%. For CHSP NPs, the methylene groups (–CH_2_CH_2_–) included two aspects: succinic anhydride and CHS. The degree of substitution was 12.34% for methylene groups (–CH_2_CH_2_–) and 7.84% for carboxyl groups. For CHAP NPs, the methylene groups (–CH_2_CH_2_–) included two aspects: ethylenediamine and CHS. The degree of substitution was 18.6% for methylene groups (–CH_2_CH_2_–) and 14.1% for the amino groups.

### Properties of CHP, CHSP, and CHAP NPs

**Fig. 4:**
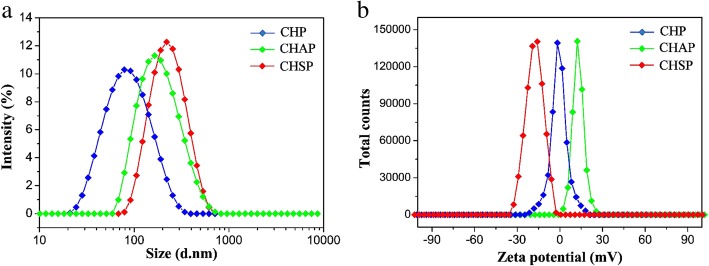
Zeta potential (**a**) and size distribution (**b**) of CHP NPs (), CHAP NPs (), and CHSP NPs ()

Amphiphilic polymers can be self-assembled by dialysis to form core–shell NPs with a hydrophilic shell and hydrophobic core, which can be loaded with anti-cancer drugs to form drug-loaded NPs. The NP properties such as surface charge and size played a vital influence on the treatment efficacy as a drug carrier [39, 40]. The size distribution and zeta potential of CHP, CHSP, and CHAP NPs measured by DLS are presented in Fig. 4. The mean size of NPs was 73.1, 116.9, and 156.9 nm for CHP, CHAP, and CHSP, respectively. The zeta potential was − 0.698, + 12.9, and − 15.4 mV, respectively (Table 1).

**Table 1:** Zeta potential and size distribution of CHP, CHAP, and CHSP NPs

Sample	D^a^ (nm)	PI^a^	ZP^b^ (mV)
CHP	73.1 ± 3.6	0.243 ± 0.015	− (0.698 ± 0.14)
CHAP	116.9 ± 2.7	0.297 ± 0.023	+ (12.9 ± 0.13)
CHSP	156.9 ± 4.1	0.299 ± 0.074	− (15.4 ± 0.18)

*PI* polydispersity index

^a^Average diameter (mean value ± SD) determined by dynamic laser light-scattering with three times

^b^Zeta potential (measured by DLS)

For pullulan NPs with the same hydrophobic group, the size was larger for negatively charged CHSP and positively charged CHAP than neutral CHP NPs. Thus, the charged group interferes with the self-assembly behavior of NPs, forming larger sized NPs with a loose structure in aqueous solution. The zeta potentials of CHP NPs, CHAP, and CHSP NPs were − 0.698 mV, + 12.9 mV, and − 15.4 mV, respectively. Thus, the amino and carboxyl groups in the polymer could create NPs with different surface charges and thus change surface properties.

### Thermodynamic Analysis

**Fig. 5:**
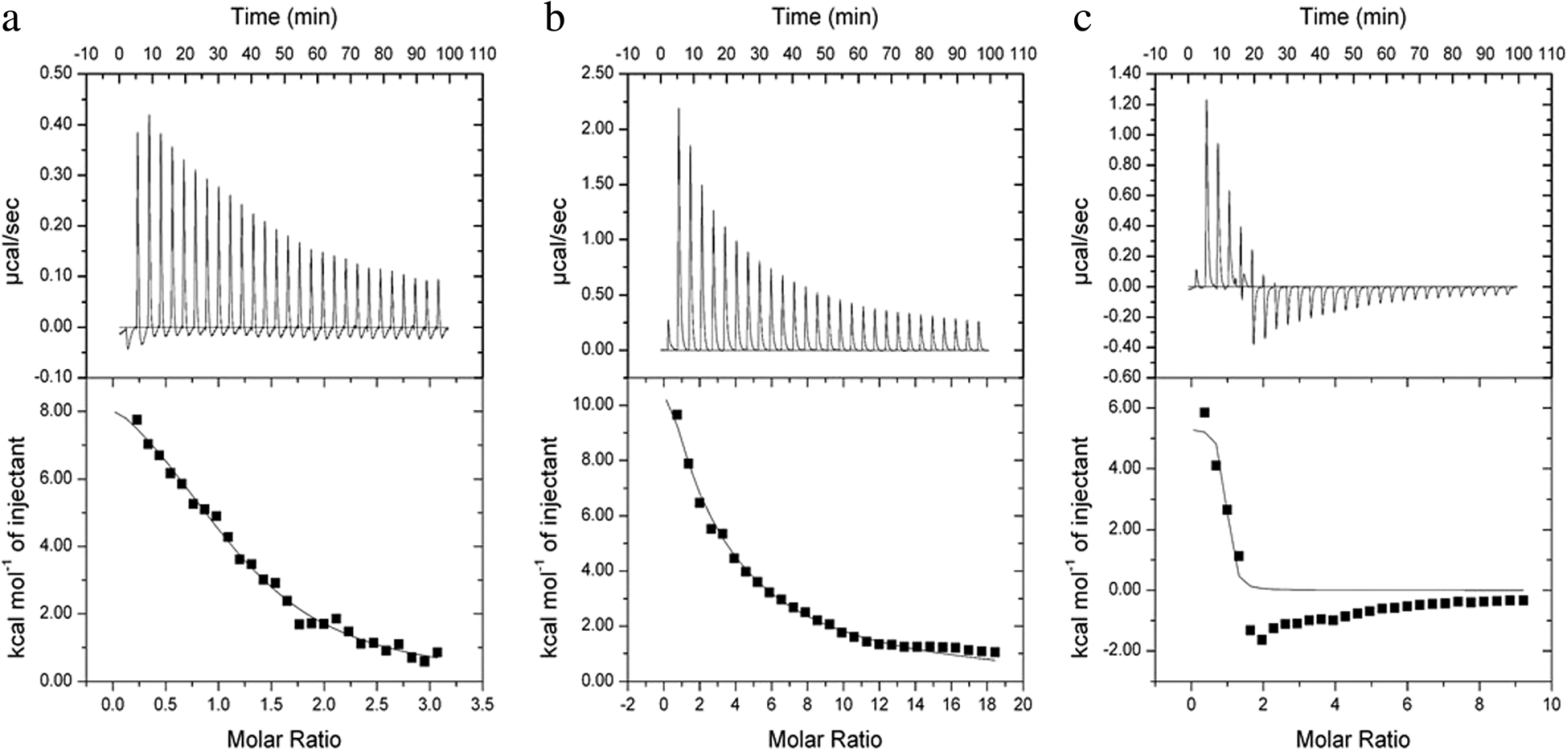
Isothermal titration calorimetry data for HSA titration into **a** CHP, **b** CHSP, and **c** CHAP NPs at 25 °C. NP concentration in the cell (250 μL) was 12 μM and the protein concentration in the syringe was 230 μM. Upper graphs show raw data, and lower graphs show integrated heats

The thermodynamic analysis was performed using ITC with HSA titrating to CHP, CHSP, and CHAP NP solutions. As HSA titration to pullulan NP solution with different charges, we determined the changes of heat, fitting out the connection properties of three kinds of materials, connection, and connection number of molecules. The original spectrum of the isothermal titration thermometer reflects the change in heat. The upward peak indicates a heat-releasing reaction, and the downward peak indicates a heat-absorbing reaction [41, 42]. When CHP, CHAP, and CHSP NPs were titrated with HSA, the released heat from the combination gradually decreased over time (Fig. 5). Overall, 26 drops of HSA solution were titrated into CHP NP solution, and the peaks of the spectrum were upward, indicating the exothermic nature of the reaction. The same phenomenon was observed when the HSA solution was titrated into the CHSP NP solution. However, when the HSA solution was titrated into the CHAP NP solution, the peaks of the spectrum for the first four drops were upward, while from the fifth drop, the peaks turned to downward, indicating an endothermic reaction. The HSA absorption of CHP and CHSP NPs was exothermic, so the reaction was spontaneous. The HSA absorption of CHAP NPs was partially endothermic and partially exothermic, which may be related to the positive charge of CHAP.

The enthalpy value reflects the heat released by the combination of HSA and NPs. The enthalpy changes of CHP, CHSP, and CHAP NPs by HSA were 42.827, 80.3712, and 22.3951 KJ/mol, respectively (Table 2). Combined with the enthalpy change, the exothermic reaction, the chemical structure of the amphiphilic NPs, and the negative charge of HSA, the hydrophobic interaction may mainly drive the HSA interaction with CHP and CHSP NPs. Of note, in the HSA titration of CHAP, the heat included a negative value. HSA molecules contain a hydrophobic pocket that can be combined with the hydrophobic center of the NP [43], so the interaction triggered by the first four drops of HSA and CHAP are primarily driven by hydrophobic forces. Although HSA is negatively charged and CHAP is positively charged, starting from the fifth drop, there is also a charge force between HSA and CHAP because of the endothermic nature of the reaction.

**Table 2:** Degree of coverage, affinity (*K*_A_), and enthalpy and entropy changes for binding with HSA titrated into NP solutions of different surface charge at 25 °C

Sample	Coverage	*K*_A_ (10^4^ M^−1^)	ΔH (KJ/mol)	ΔS (KJ/mol K)
CHP	1.17 ± 0.0831	27.7 ± 3.26	43.827 ± 2.353	0.251
CHSP	0.404 ± 0.0435	1.41 ± 0.199	80.3712 ± 16.84	2.775
CHAP	0.845 ± 0.0922	412 ± 9.16	22.395 ± 3.949	0.201

The value of entropy change can reflect the difficulty of the reaction between HSA and NPs. The entropies of HSA and CHP, CHSP, and CHAP NPs were 0.251, 2.775, and 0.201 KJ/mol K, respectively (Table 2), so CHP and CHAP NPs are more easily to bind to HSA, while CHSP NPs are more difficult to bind to HSA.

The coverage of HSA and CHP, CHSP, and CHAP NPs was 1.17, 0.404, and 0.845, respectively. The concentration of NPs is calculated based on the concentration of the polymer rather than the number of particles. We do not know how many single polymer particles contain a single NP, so we cannot determine exactly how much of each NP is adsorbed by HSA, but based on the value of coverage, we can conclude that CHP NPs have the highest adsorption of HSA.

In previous experiments, we showed that the affinity value, *K*_A_, reflects the strength of the binding force between NPs and HSA [32]. The stronger the hydrophobicity of CHP NPs, the stronger the affinity [44]. The binding constant of HSA and CHP was 27.7 × 10^4^ M^−1^, that of HSA and CHSP was 1.41 × 10^4^ M^−1^, and that of HSA and CHAP was 412 × 10^4^ M^−1^. So, the combination of HSA and positively charged CHAP was the strongest, followed by neutral-charged CHP and negatively charged CHSP. The strongest combination of HSA and CHAP may be attributed to the hydrophobic force, a mutual attraction of the charge force and probably a mutually exclusive charge between HSA and CHSP.

### Fluorescence Spectroscopy

**Fig. 6:**
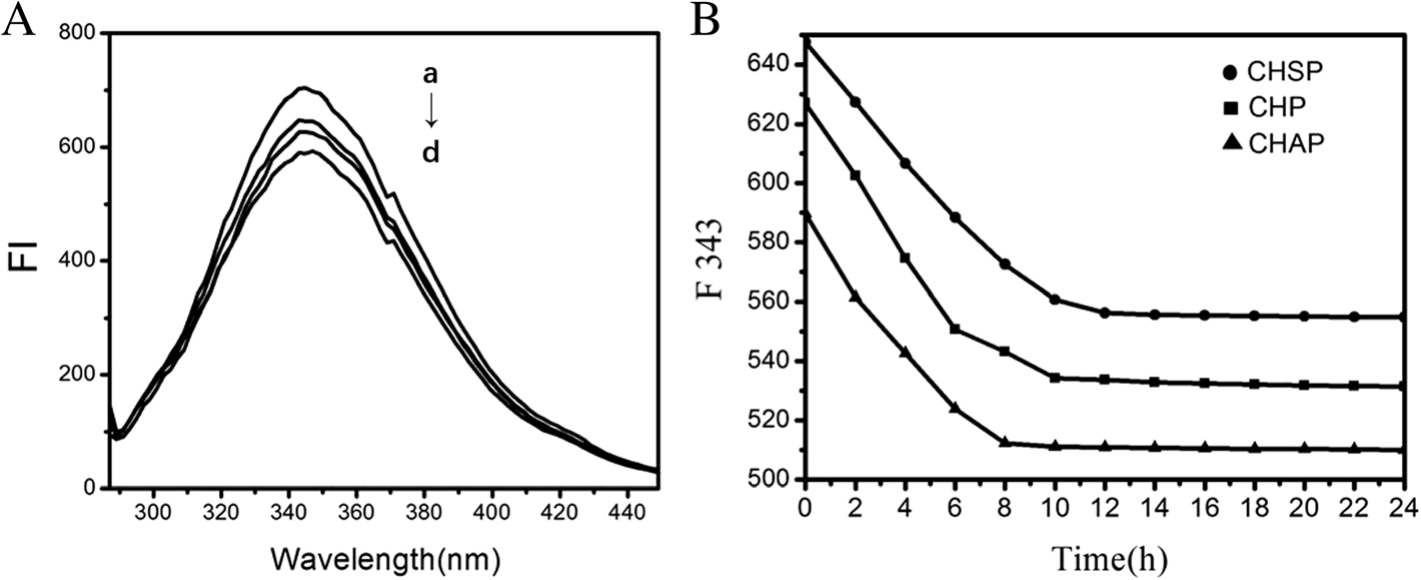
**A** Fluorescence spectra for HSA (1.5 × 10^− 5^ mol/L) without (a) and with (b) CHSP, (c) CHP and (d) CHAP NPs with the same concentration (4.2 × 10^− 6^ mol/L). **B** HSA emission intensity with CHSP, CHP, and CHAP NPs at 343 nm over time

With fluorescence spectra, we studied the interaction between HSA and the three NPs with different surface charges. HSA contains 585 amino acid residues, with only one Trp residue at 214 (Trp214), and its fluorescence spectrum dominates in the UV region. When other molecules interact with HSA, the fluorescence spectrum of Trp may change, depending on the interaction between HSA and other molecules. When the three NPs were mixed with HSA, the maximum emission peak of the HSA fluorescence spectrum did not undergo a chemical shift; only the intensity was weakened to a certain extent (Fig. 6A). The maximum emission peak of HSA was observed when HSA combined with CHAP NPs.

Experimental study showed that combining HSA and pullulan NPs displayed a complexed process [45]. We added the three NPs into HSA solution and found that the fluorescence intensity of the three NP–HSA complexes decreased gradually (Fig. 6B). The equilibrium was reached for HSA–CHSP at 556.3 nm after 12 h, for CHP–HSA at 534.3 nm after 10 h, and for CHAP–HSA at 512.3 nm after 8 h. The required time when the fluorescence intensity of different NP–HSA complexes is balanced is related to the charge carried by the NP. The longest time required to reach equilibrium was observed on negatively charged CHSP–HSA, followed by uncharged CHP–HSA.

The rapid reduction in initial fluorescence intensity of the three NP–HSA complexes (shown in Fig. 6A) is due to the rapid adsorption of NPs and HSA. The subsequent fluorescence intensity shows a slow decrease to the constant status because the interaction of NPs with HSA is a slow process of combination. The constant fluorescence intensity reflects the saturation status of the complexation. The interaction between the three different-charged NPs and HSA underwent an early rapid recombination process and a later slow recombination process. The combination of negatively charged HSA with negatively charged CHSP required a longer time to achieve complex saturation compared with uncharged CHP. Negatively charged HSA combining with positively charged CHAP required the shortest time to reach complex saturation.

**Fig. 7:**
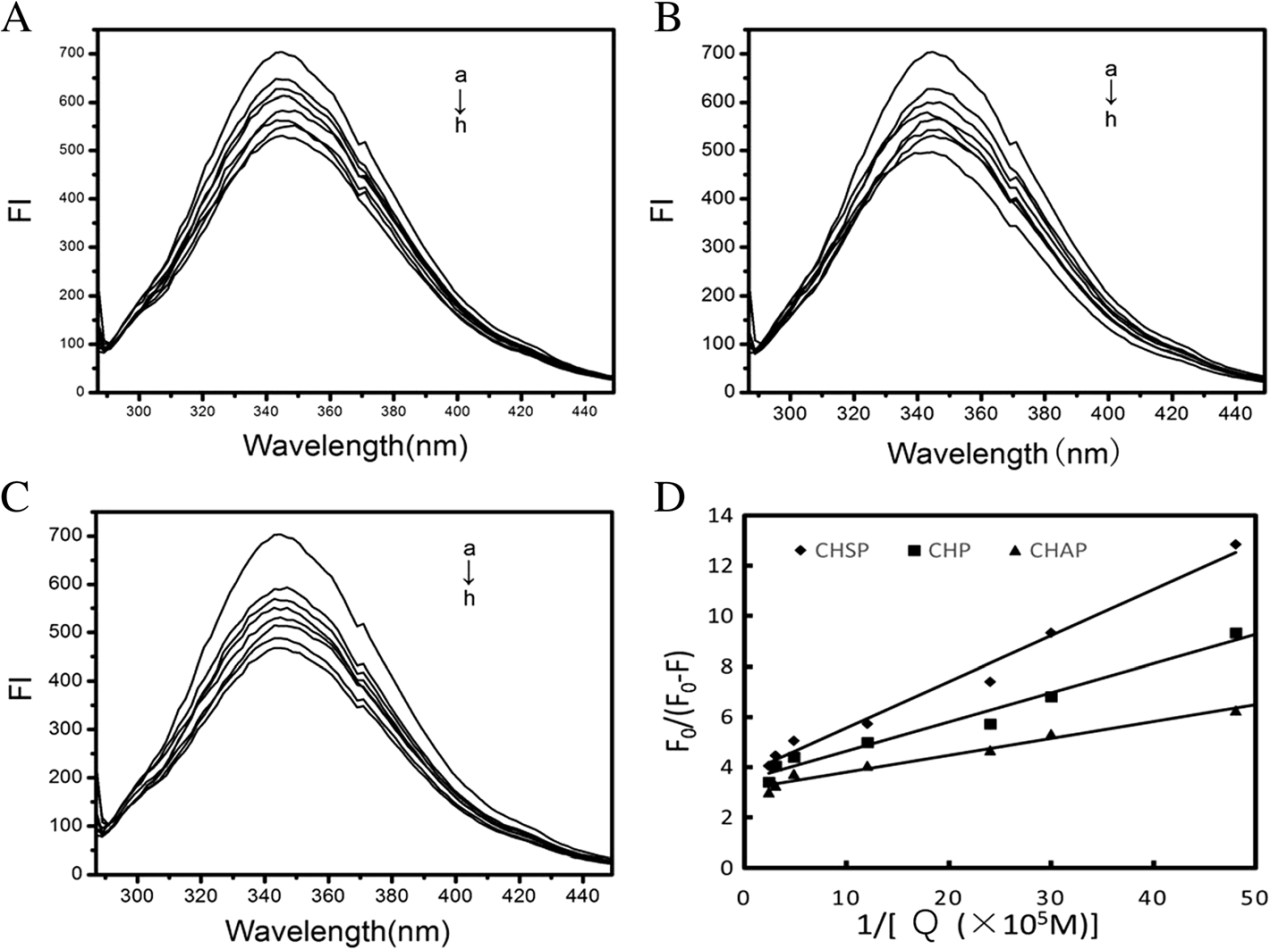
Fluorescence spectra of HSA (1.5 × 10^− 5^ mol/L) with CHSP (**A**), CHP (**B**), and CHAP (**C**) at different concentrations (a) 0, (b) 2.07 × 10^−7^, (c) 3.31 × 10^−7^, (d) 4.14 × 10^−7^, (e) 8.28 × 10^−7^, (f) 20.7 × 10^−7^, (g) 33.1 × 10^−7^, and (h) 41.4 × 10^−7^ mol/L. **D** Plots (*n* = 7) for *F*_0_/(*F*_0_ − F) vs 1/[*Q*], *Q* is the concentration of CHSP (–◆–), CHP (–■–) and CHAP (–▲–), respectively

Figure 7A–C shows the spectra of HSA combined with different concentrations of CHSP, CHP, and CHAP NPs to form NP–HSA complexes. With increasing concentration of NPs, the maximum absorption peak of the NP–HSA complex decreased showing a reverse correlation.

The positively charged CHAP-HSA displayed the shortest time to reach equilibrium.

We used a modified Stern–Volmer equation to analyze the fluorescence quenching data:

$$ {F}_0/\left({F}_0-F\right)=1/{f}_{\mathrm{a}}+1/\left({f}_{\mathrm{a}}{K}_{\mathrm{q}}\left[Q\right]\right) $$

where *f*_a_ is the contact fraction of the fluorescent substance with the quencher, *K*_q_ is the Stern–Volmer quenching constant, *F*_0_ is the fluorescence intensity at 342 nm without the quencher, *F* is the fluorescence intensity at 342 nm with the quencher, and [*Q*] is the concentration of quencher.

From the functional image of *F*_0_/(*F*_0_ − *F*) pair 1/[*Q*], we can obtain the values of *f*_a_ and *K*_q_ from the slope and the intercept (Fig. 7D). Assuming that the observed fluorescence intensity changes mainly due to the interaction between NPs and HSA, the quenching constant can be seen as a binding constant for the formation of complexes. The binding constants for HSA and CHSP, CHP, and CHAP molecules were 2.02, 2.99, 4.72 × 10^5^ M^−1^, respectively. In the previous study, we discussed the interaction between HSA and CHP NPs with different hydrophobicity substitutions [30]. The hydrophobic interaction between HSA molecules and CHP cholesterol played an important role in the formation of CHP–HSA. The greater the hydrophobic substitution of CHP, the greater the binding constant of CHP and HSA.

In the present study, we found that the value of the binding constant (*K*_b_) is related to the electrical properties of the NP. A surface with a positive charge, such as CHAP, has the largest *K*_b_, and a surface without a charge, such as CHP, has the second largest *K*_b_. The *K*_b_ for CHSP, with a negative charge, was the lowest. Therefore, in addition to the hydrophobic interaction between NPs and HSA, the electrostatic interaction between them also plays an essential part in the formation of NP–HSA complexes.

In addition, the *f*_a_ values for CHSP, CHP, and CHAP NPs were 0.269, 0.288, 0.38, respectively, so part of the Trp residue was involved in this reaction. Furthermore, the amount of HSA was too much at a given NP/HSA concentration, so free HSA molecules presented in the reaction system.

### CD Spectrum Analysis

**Fig. 8:**
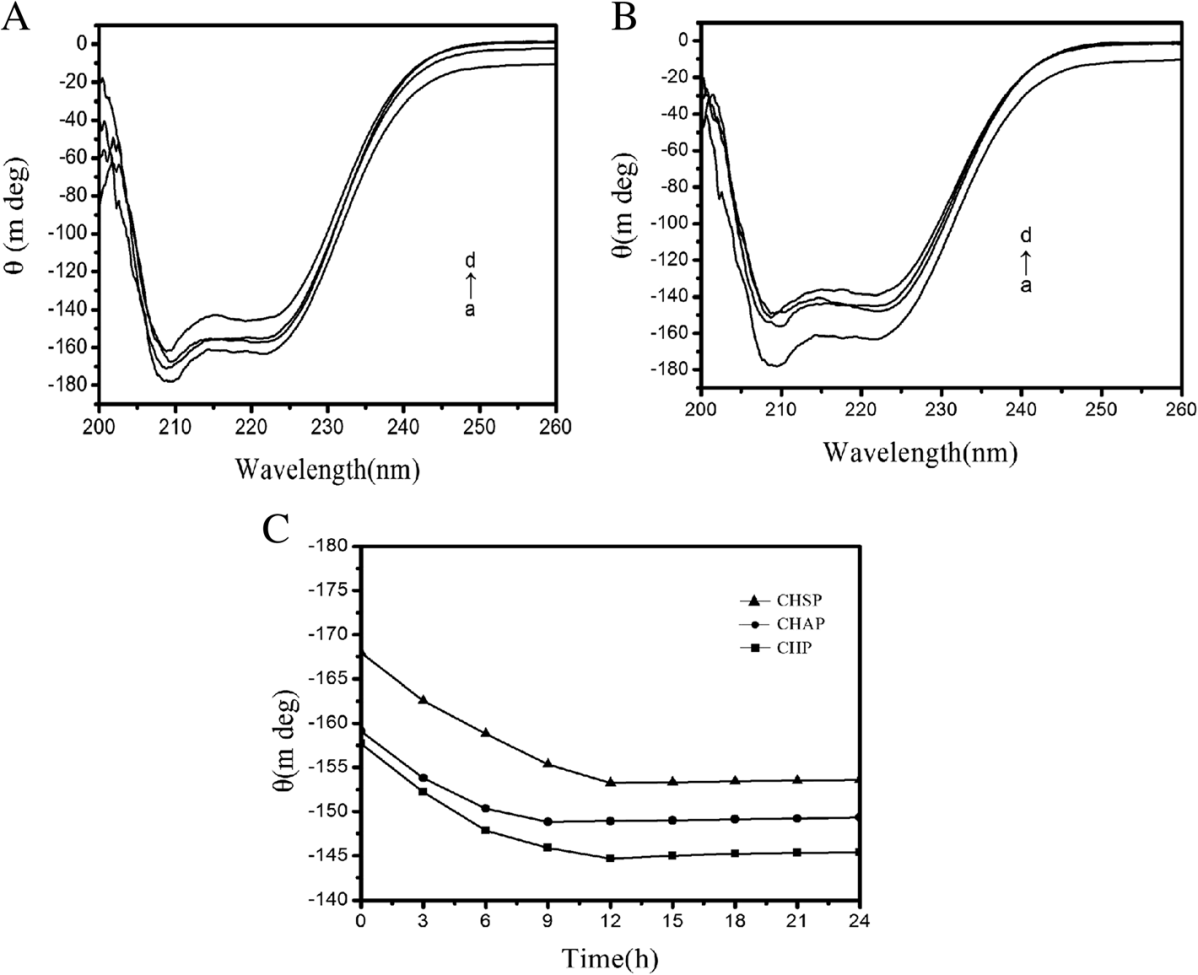
**A** CD spectra for (a) free HSA, (b) CHSP–HSA, (c) CHAP–HSA, and (d) CHP–HSA in solution at 25 °C. The samples were complex I. **B** CD spectra for (a) free HSA, (b) CHSP–HSA, (c) CHAP–HSA, and (d) CHP–HSA in solution at 25 °C. The samples were complex II. **C** Ellipticity at 208 nm for HSA interacting with CHSP (–▲–), CHAP (–●–) and CHP (–■–) over time

Figure 8A shows the CD spectra (a) free HSA, (b) CHSP–HSA, (c) CHAP–HSA, and (d) CHP–HSA in solution at 25 °C. The samples were complex I. There are two negative bands at 208 and 222 nm of the UV region in HSA spectra, which are the characteristic peaks of the *α*-helical structure. The *α*-helical content of free HSA was 55%. At the beginning of the complex, with the recombination of HSA and CHSP, CHAP, and CHP NPs, the *α*-helical content of HSA was reduced to 52.0%, 48.57%, and 48.0%, respectively.

Figure 8C shows that the ellipticity changes of the three samples at 208 nm over time. The ellipticity of HSA combined with CHAP and CHP NPs increased from 0 to 12 h and leveled off at 12 h, but that of HSA with CHSP NPs increased faster, i.e., the ellipticity increased from 0 to 9 h and leveled off at 9 h. Therefore, the ellipticity of the three samples gradually increased over time and the α-helical content gradually decreased; when the ellipticity maintained constant, the recombination of the sample and HSA was completed.

Figure 8C illustrates that the fastest surface absorption rate was presented on the combination of CHSP and HSA. The size, charge, and hydrophobicity of NPs can influence the migration rate of HSA to the center of NPs. The surface of CHP is not charged, the surface of CHSP is negatively charged, and the surface of CHAP is positively charged. Owing to the presence of the negative-charge mutual exclusion between CHSP and HSA, the resistance of HSA migrating to the CHSP NP center becomes larger. The traction of NPs on HSA is driven by the hydrophobic interaction forces. The traction of the three NPs on HSA is identical, so the lowest velocity of HSA migrating to the center of NPs was observed on the combination of HSA and CHSP NPs. Because the particle size is in the order of CHSP > CHAP > CHP, the particle density displays a reverse order, i.e., CHSP < CHAP < CHP. In principle, the speed that HSA migrates toward the NP center is fastest for CHSP NPs and slowest for CHP NPs.

The ITC results show that the amount of HSA migrating toward the center of CHSP NPs is the smallest but with the fastest speed compared with other types of NPs. Table 3 shows that the α-helix content of HSA migrating toward the center of CHP NPs was the lowest. The more the secondary structure of HSA was damaged, the faster the HSA migrated toward the center. The fastest speed was observed on HSA migrating to the center of CHSP NPs (Fig. 8C).

**Table 3:** The α-helical contents of three samples

Samples	CHP–HSA (%)	CHAP–HSA (%)	CHSP–HSA (%)
Complex I	48.01	48.57	52.04
Complex II	42.91	44.55	46.27

Figure 8B shows CD spectra for (a) free HSA, (b) CHSP–HSA, (c) CHAP–HSA, and (d) CHP–HSA in solution at 25 °C. The samples were complex II. After the complexation is completed, the *α*-helical content of HSA recombined with CHSP, CHAP, and CHP was reduced to 46.27%, 44.55%, and 42.91%, respectively (Table 3). With the increase in interaction time, the secondary structure of HSA was changed and the *α*-helical content was reduced during the process of complexation with NPs.

### Drug Release

**Fig. 9:**
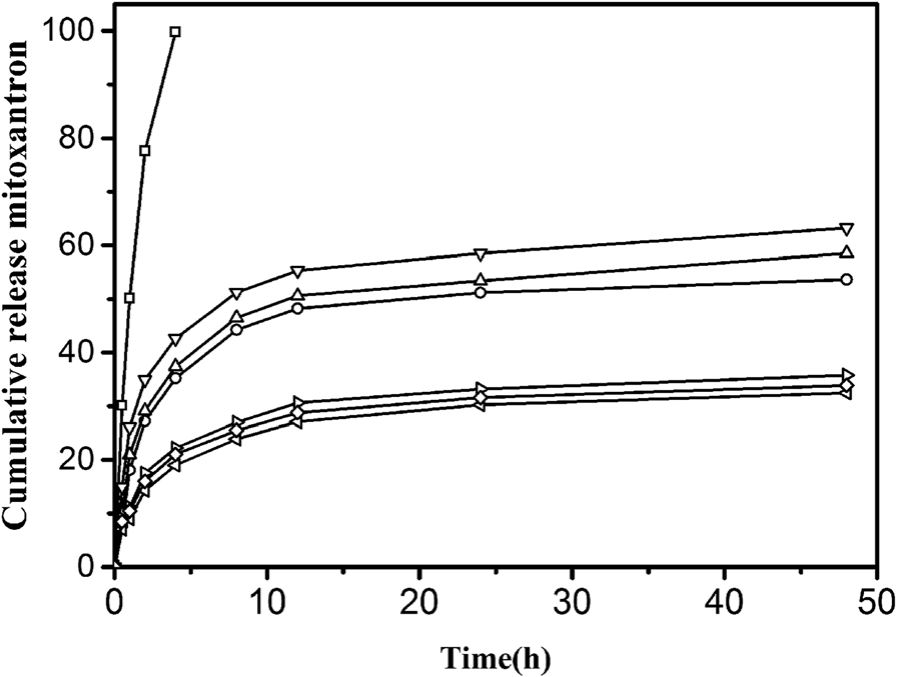
Mitoxantrone (MTO) release of pullulan NPs in phosphate-buffered saline (PBS) at 37 °C in vitro (□: free mitoxantrone, ○: CHP, △: CHAP, ▽: CHSP, ◁: CHAP–HSA,◇: CHP–HSA, ▷: CHSP–HSA)

We measured the drug release rate of MTO with the three kinds of drug-loaded NPs and drug-loaded NP–HSA complexes. The drug release for free MTO was about 99.8% at 4 h (Fig. 9). The release rate in 48 h for CHP, CHAP, and CHSP NPs was 53.68%, 58.54%, and 63.24%, respectively. The drug release from all NPs was fast in the first 8 h, which was a burst release process, and the drug release remained stable after 12 h, which was a sustained release process. In vitro drug release from NPs is not affected by gastrointestinal pH and enzymes, and the dissolution of nanomaterials results in little drug release. The release is mainly determined by dissolution and diffusion [46]. The 48-h drug release rate was in the order of CHSP > CHAP > CHP, corresponding to the size of NPs. The fastest release rate was found on CHSP, which was negatively charged with the largest size. The second fast drug release rate was found on which was positively charged with the size smaller than CHSP. CHP was electrically neutral, and its drug release rate was minimal. Hence, the polymer surface groups involved in the formation of NPs affect the size of NPs and ultimately the drug release of NPs.

The drug release rate from the combinations of HSA and CHAP, CHP, and CHSP in 48 h was 32.45%, 33.86%, and 35.76%, respectively. After the combination of NPs and HSA, the drug release in 48 h was significantly decreased as compared with HSA-free NPs, which was mainly attributed to the resistive effect and adsorption effect of HSA. At 48 h, the drug release of the compounds was in the order of CHSP–HSA > CHP–HSA > CHAP–HSA, while the drug release of HSA-free NPs was in the order of CHSP > CHAP > CHP. Although NP–HSA compounds showed significantly slow drug release, the total release of CHP-HSA decreased by 21.23% in 48 h, whereas the total release of CHAP-HSA decreased by 25.68% and that of CHSP-HSA by 28.48%. The drug release of the NPs is related to the size of the NPs and the polymer hydrophobic groups on NP surface. The adsorption of HSA can lead to significant slowdown in drug release, which is related to the hydrophobicity of NPs and also to the surface charge of NPs [46]. The adsorption of HSA is closely related to the size of the NPs and the degree of substitution of the hydrophobic groups of the polymer during the self-assembly process. Nevertheless, the drug release of the NPs is ultimately determined by the properties of the NP itself.

## Discussion

**Fig. 10:**
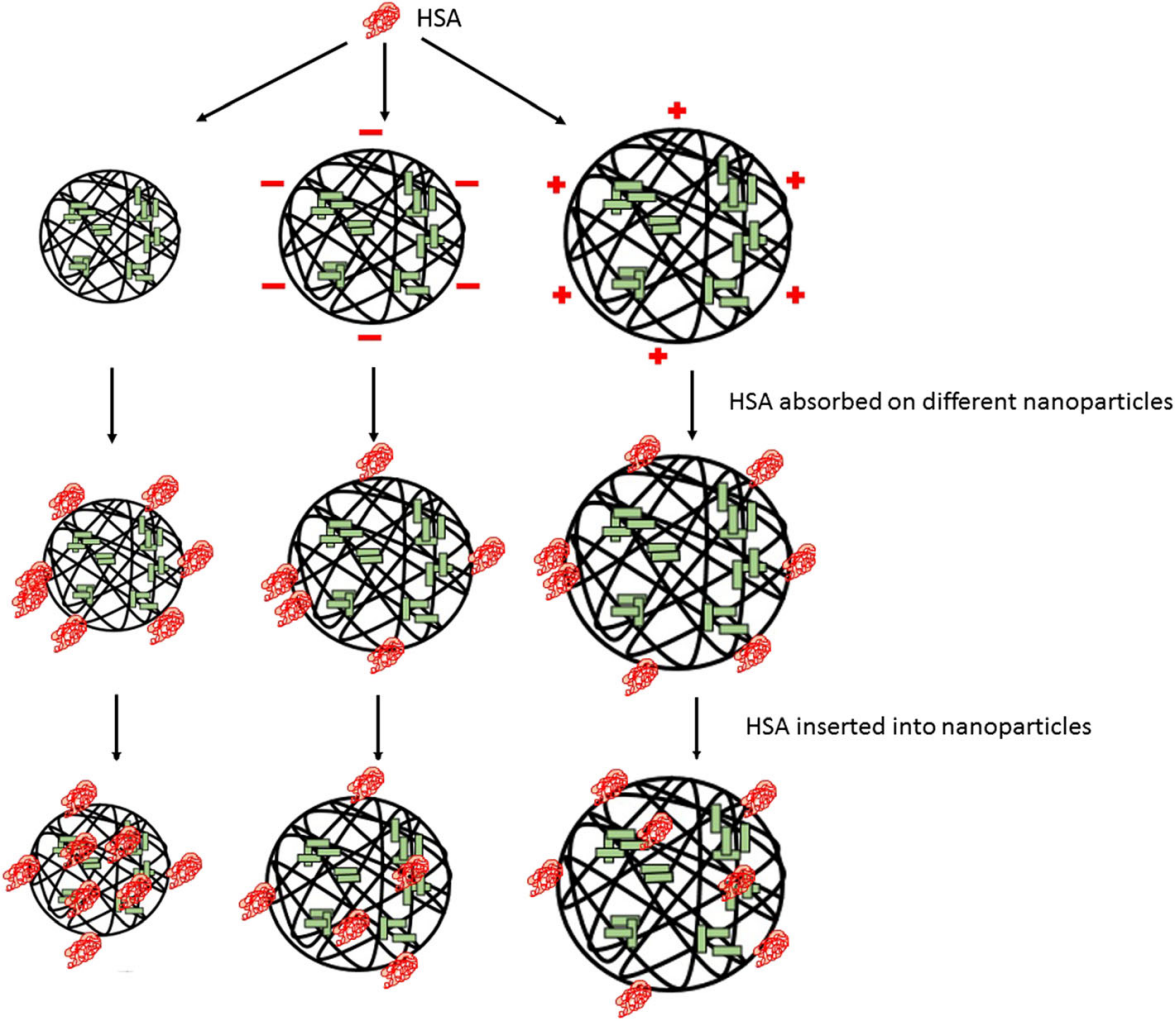
Adsorption of HSA to NPs

As shown in Fig. 10, the formation of the NP–HSA complex is driven by a hydrophobic force between cholesterol groups of the particle core and the aromatic amino acid of the hydrophobic domain of HSA. After mixing, HSA interacts with the surface cholesterol unit and is rapidly adsorbed to the NP surface. Then, the adsorbed HSA on the NP surface is processed because of the hydrophobic forces derived from the cholesteric unit in the particle core. When overcoming the steric hindrance of polysaccharide chains in the NP shell, the adsorbed HSA gradually migrates to the core. After the hydrophobic interaction and resistance of the hydrophilic polysaccharide chain are balanced, the HSA molecule enters the particle core to become hydrophobically bound to cholesterol groups to form the NP–HSA complex.

For CHAP and CHSP NPs, the recombination of HSA is a complex process also subjected to the charge interaction with HSA under the traction of the hydrophobic driving force. The binding constants of the three kinds of NPs with the same hydrophobic substitution and different surface charge were in the order of CHAP > CHP > CHSP. The electrical properties also play a major part in the formation of NP–HSA. In this process, the formation of the CHSP–HSA complex was blocked by the structure of the NP shell and the repulsive force between the negative charges, which led to their loose connection. During the rapid adsorption and slow recombination, the degree of spiraling of HSA is lower for CHSP NPs than CHP NPs and CHAP NPs. Therefore, the surface charge of NPs not only changes the nature of the particles themselves but also affects the protein complex.

In the current study, we investigated the effect of NP surface charge on the interaction between NPs and proteins (Fig. 10). Three different charges of pullulan NPs with HSA adsorption still showed rapid adsorption and slow recombination. The number of HSA molecules with positively charged CHAP complex was the most, including rapid adsorption of NP–HSA by hydrophobic forces, HSA molecule migration to the center, and the HSA molecules adsorbed on the surface of NPs by charge action. CHP- and CHSP-adsorbed HSA molecules were mainly distributed in the hydrophobic center of NPs, with CHSP adsorbing fewer HSA molecules. The adsorbed number of HSA molecules is related to the hydrophobicity of NPs. The greater the degree of substitution of hydrophobicity, the more HSA is adsorbed [41]. The cholesterol substitutions of the three NPs were the same, and the number of HSA molecules adsorbed by positively charged NPs was the highest, so the adsorption of NPs and HSA was related to the hydrophobicity and surface charge of the NPs.

The surface adsorption capacity between NPs and HSA is also related to the hydrophobicity and charge of NPs. The binding force between HSA and NPs is determined by the hydrophobicity, surface charge, size, and structure of NPs. The α-helicity was decreased most at the beginning of adsorption and the complete CHP–HSA complex. CHP NP has the smallest size and highest density. The CHP NPs migrated toward the center by the hydrophobic traction; the sugar chain of the CHP NP shell was larger to inhibit the migration toward the center. The extension of the peptide chain of HSA is larger, with the *α*-helix decreased the most. Although CHAP NPs have hydrophobic and charge forces, they possess relatively large size, loose structure, small resistance in the periphery, small extension of the peptide chain, and small content of the *α*-helix. Some HSAs remained on the surface of NPs through the charge force of adsorbing, and the *α*-helical content is also smaller in this part of the HSA. The *α*-helix content of CHAP decreased less than that of CHP, mainly due to the peptide chain extension-induced central pulling force which led to α-helix content decline. During the process of the CHSP and HSA complexation, the role of the central pulling force has a reverse direction of the charge force, thereby resulting in weakening the center of the migration force. CHSP NPs are larger than CHAP NPs, and the structure of CHAP NPs is loose. Because the adsorbed number of HSA on CHAP is higher than that on CHSP, the decrease of *α*-helicity in CHSP is less than that in CHAP NPs. Therefore, the interaction between NPs and HSA and the decrease in *α*-helicity are all related to the size, density, hydrophobicity of substitution, surface charge of the NPs, and number of HSA connections.

After the NPs enter into the blood, protein adsorption affects the functions of NPs, such as the slow and controlled drug release, the travel from the blood circulation passing through the vascular barrier, targeting tissue, and entering cells. NPs interact with the HSA in the body and affect the in vivo behavior of NPs. The number of adsorbed proteins is closely related to the properties of the NPs. HSA adsorbs NPs, which affects the distribution in organs and removal of NPs, thereby altering the concentration of the drug in the body and the efficacy of the drug.

Finally, the properties of NPs, such as size, hydrophobicity, and surface charge, affect the drug release of NPs in vivo. We can design specific materials to perform specific functions with specific protein adsorption.

## Conclusions

In this study, three kinds of nano-drug carriers were constructed, CHP, CHSP, and CHAP. The size, charge, drug loading properties of NPs, interaction between NPs and HSA, and drug release were all closely related to charge amount and charge type of nanomaterials. With the same degree of substitution of hydrophobicity, CHAP NPs with larger amino substitutions were the largest, CHSP NPs the second largest, and CHP NPs the smallest. The size and surface charge of the NPs were essential to the coverage of HSA, the binding constant, and the slow drug release. The positively charged CHAP binding constant was the strongest, showing the fastest drug release, and CHP NPs had the highest coverage. The combination of HSA further retarded the drug release of NPs. CHAP NPs adsorbed HSA had the slowest drug release rate.

## Abbreviations

CH: Cholesteric hydrophobically
CHAP: CH-modified animated pullulan
CHP: Cholesteric hydrophobically (CH) modified pullulan
CHSP: CH-modified carboxylated pullulan
DMSO: Dehydrated dimethyl sulfoxide
HSA: Human serum albumin
*K*_b_: The binding constant
MTO: Mitoxantrone
NP: Nanoparticle
